# Pyrolysis and Physicochemical, Thermokinetic and Thermodynamic Analyses of *Ceiba aesculifolia* (Kunth) Britt and Baker Waste to Evaluate Its Bioenergy Potential

**DOI:** 10.3390/molecules29184388

**Published:** 2024-09-15

**Authors:** José Juan Alvarado Flores, Luis Fernando Pintor Ibarra, Fernando Daniel Mendez Zetina, José Guadalupe Rutiaga Quiñones, Jorge Víctor Alcaraz Vera, María Liliana Ávalos Rodríguez

**Affiliations:** 1 Facultad de Ingeniería en Tecnología de la Madera, Universidad Michoacana de San Nicolás de Hidalgo, Edif. D. Cd. Universitaria, Santiago Tapia No. 403, Centro, Morelia 58000, Mexico; luis.pintor@umich.mx (L.F.P.I.); 1614346b@umich.mx (F.D.M.Z.); jose.rutiaga@umich.mx (J.G.R.Q.); 2 Instituto de Investigaciones Económicas y Empresariales, Universidad Michoacana de San Nicolás de Hidalgo, Cd. Universitaria, Santiago Tapia No. 403, Centro, Morelia 58000, Mexico; jorge.alcaraz@umich.mx; 3 Centro de Investigaciones en Geografía Ambiental, Universidad Nacional Autónoma de México, Antigua Carretera a Pátzcuaro No. 8701, Col. Ex Hacienda de San José de la Huerta, Morelia 58190, Mexico

**Keywords:** pyrolysis, physiochemical properties, TGA-DTG, iso-conversional kinetic methods, thermodynamic analyses and bioenergy potential

## Abstract

*Ceiba aesculifolia* is an important species in Mexico that generates significant amounts of biomass waste during its exploitation, which can be utilized to produce energy. This study presents the characterization of this waste based on chemical (proximal and elemental) and thermal analyses (TGA-DTG) at different heating rates (β = 10–30 °C/min (283–303 K/min)) in the presence of nitrogen and in a temperature range of 25–900 °C. Kinetic parameters were calculated and analyzed as well. Activation energy (*Ea*) and the pre-exponential factor (*A*) were determined using the Friedman (132.03 kJ/mol, 8.11E + 10 s ^−1^), FWO (121.65 kJ/mol, 4.30E + 09), KAS (118.14 kJ/mol, 2.41E + 09), and Kissinger (155.85 kJ/mol, 3.47E + 11) kinetic methods. Variation in the reaction order, *n* (0.3937–0.6141), was obtained by Avrami’s theory. We also calculated the thermodynamic parameters (ΔH, ΔG, ΔS) for each kinetic method applied. The results for *Ea*, *A*, *n*, Δ*H*, Δ*G*, and Δ*S* show that this biomass waste is apt for use in pyrolysis. Moreover, the moisture (<10%), ash (<2%), volatile material (>80%), and HHV (>19%) contents of *C. aesculifolia* allowed us to predict acceptable performance in generating energy and fuels. Finally, infrared spectroscopy analysis (FT-IR) allowed us to identify important functional groups, including one that belongs to the family of the aliphatic hydrocarbons.

## 1. Introduction

**Figure 1:**
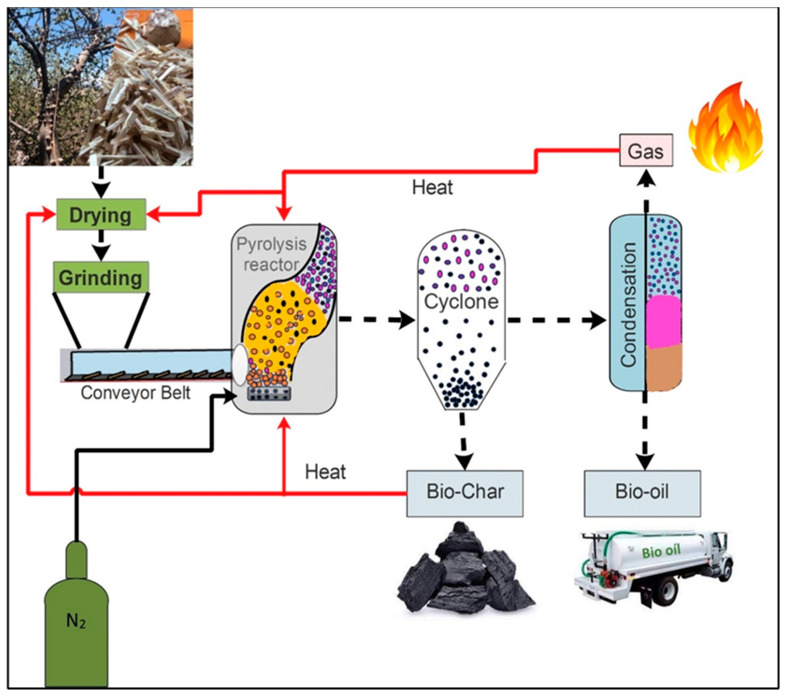
Generation of high-value-added products from biomass.

Today, one of the most attractive options worldwide for developing energies based on alternative sources is biomass. Mexico is no exception in this regard. Every day in western Mexico, thousands of tons (1,018,407.17 m^3^) of biomass is produced as lignocellulosic waste from the exploitation of forest species like pine, oak, mahogany, parotta (elephant-ear tree), and ceiba, among others [1]. This waste consists mainly of sawdust, branches, leaves, bark, and stumps. Unfortunately, it is rarely used for purposes other than as fuel for cooking or drying wood, or in ovens to cook adobe bricks [2]. However, research into the energy potential of biomass waste, focused on utilizing it to produce fuels that could eventually replace fossil fuels, has expanded around the world [3]. Biomass waste from *Ceiba aesculifolia* (Kunth) Britt and Baker (hereinafter, *C. aesculifolia,* known locally as pochote) is an option that may help achieve this goal. This tree, which grows in western Mexico, including the state of Michoacán, is notable for its natural ability of constant regeneration. It is well known that lignocellulosic biomass—terrestrial and marine—can be thermally transformed to generate energy. Currently, thermochemical processes (pyrolysis, gasification, combustion) are viable options for converting biomass into products with high added value, such as bio-oil, bio-char, and combustible gas (Figure 1), all of which can be used to generate electrical energy by means of fuel cells [4,5].

Of the processes mentioned above, pyrolysis is one of the most widely used forms of thermochemical transformation of biomass waste not only into lignocellulosic biomass, but also into animal biomass waste, such as manure [6], and even in materials such as microplastics [7]. This thermal process, also called destructive distillation, is carried out in an inert atmosphere (e.g., nitrogen and argon), in contrast to the process that requires oxygen (complete combustion). Pyrolysis of biomass can be used to obtain several substances of importance for industry, such as oil, coal, hydrocarbon chains (e.g., methane), aromatic compounds, and hydrogen (H_2_), known as syngas, which is usually obtained industrially through petroleum refining processes. Pyrolysis generally requires temperatures of 400–600 °C and a pressure of 1–5 bar [8]. Given the efficiency of hydrogen, the possibility of obtaining this gas from biomass, and its potential for generating clean, renewable, environmentally friendly electrical energy, numerous researchers have taken up the challenge of optimizing processes to obtain it. Published studies show good average yields of 55, 40, 48, 50, 48, and 50% H_2_ in biomass derived from nogal and cotton, and waste from tea, olive, beech, and fir production, respectively [9]. Significantly, studies of biomass include marine residues like algae [10]. At the time of our study, however, there were no reports on the biomass waste of *C. aesculifolia*. 

It is well known that, thanks to its physicochemical properties and lignocellulosic composition, biomass is an excellent option as an energy source. One key aspect in optimizing yields consists of analyzing the kinetic parameters of the thermochemical conversional process utilized (in the present case, pyrolysis). This study documents the chemical, energy, and thermal characterization of this material and discusses the handling of the data obtained from thermogravimetric analyses and their application in well-known mathematical methods that make it possible to optimize the energy extracted from this biomass. We applied three iso-conversional methods—Friedman, Flynn–Wall–Ozawa (FWO), and Kissinger–Akahira–Sunose (KAS)—and Kissinger’s non-iso-conversional type to obtain precise values for three kinetic parameters—apparent energy activation, *Ea*, the pre-exponential factor, *A*, and the reaction order, *n*—as well as the Gibbs free energy change as a thermodynamic parameter, and changes in enthalpy and entropy. In addition to the fact that this thermokinetic study is the first to be carried out for *C. aesculifolia*, we believe that it is necessary to make better energy use of the enormous amounts of biomass waste such as sawdust (more than 2000 kg) and shavings (more than 5000 kg) that are generated per week in some communities in Mexico [11].

## 2. Results and Discussion

### 2.1. Determination of Basic Density 

The basic density of wood is a physical characteristic that has a crucial role in processes of biomass combustion [12,13]. Studies of this factor show that an increase in basic density leads to an increase in calorific value; that is, a larger amount of energy stored per unit of volume. According to the literature [14], *C. aesculifolia* wood is classified as “heavy”, with a basic density of 0.70 g/cm^3^. For the wood of the group of leafy trees, basic density varies from 0.475 to 0.814 g/cm^3^ [15], so *C. aesculifolia* is within this range.

### 2.2. Basic Chemical Composition

#### 2.2.1. Holocellulose, Cellulose, and Hemicellulose

**Figure 2:**
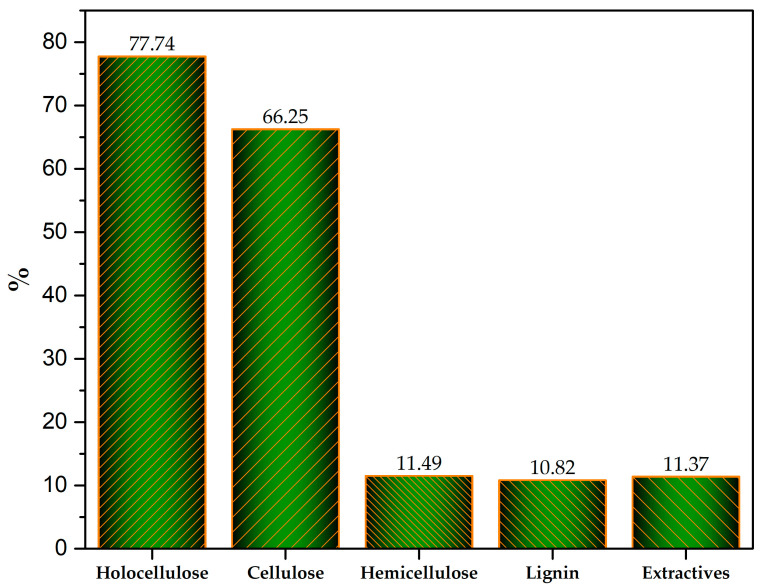
Basic chemical composition of *C. aesculifolia* wood.

Holocellulose constitutes the total fraction of the polysaccharides present in wood, including cellulose (CE) and hemicelluloses (HMs) [16,17]. In this study, the sum of CE and HM resulted in a total of 77.74%, a percentage above those reported for other hardwoods in Mexico, which tend to fall between 63.8 and 73.58% [18,19]. It is important to clarify that the total content of the basic composition shown in Figure 2, practically 100%, is only the sum of EC, HMs, lignin, and extractables.

The cellulose content of *C. aesculifolia* wood (see Figure 2) exceeds the 41.29% reported for *Ceiba pentandra* [20]; in fact, the value obtained herein is above the range of 41.32–58.83% registered for Mexican hardwoods [21]. This suggests its potential for use in producing bioethanol, although the CE is found mainly in crystalline form, which can impede its depolymerization for fermentation due to problems of accessibility and the consumption of chemical reagents [22]. This high percentage (66.25%) means that *C. aesculifolia* wood can be effective in obtaining CE destined for producing paper and its derivatives, a process that is expected to give high yields [23]. The percentage of hemicelluloses (11.49%) can be considered within the range (11.75–24.38%) found in five hardwood species native to Mexico that have characteristics similar to those of the biomass used in this study [24]. It is important to mention that the HMs present in amorphous regions are more easily hydrolyzed using enzymes than the crystalline forms, so they could be useful in fermentation processes [25].

#### 2.2.2. Lignin

Lignin is the second-most-abundant amorphous biopolymer in nature. Its principal derivatives are coniferyl, coumaryl, and sinapyl alcohol. Lignin represents 15–30% of the composition of wood [26]. We found that *C. aesculifolia* contains 10.82% lignin (Figure 2), although other authors have recorded percentages from 14.86 to 36.57% in Mexican hardwoods [19]. Clearly, the value found in our work is below this range—a fact that could favor bioethanol production, since lignocellulosic materials with high percentages of lignin can impede fermentation treatments [22]. High lignin content in wood, in contrast, can improve its quality as a solid biofuel, because this compound increases the higher heating value [27,28].

#### 2.2.3. Extractives

In general, extractives are organic substances of low molecular mass that are not part of the wood cell wall. They are also commonly called accessory substances or extraneous components, are formed from the secondary metabolism of plants, are removed by water and organic solvents (water, alcohol, acetone, benzene, ether, and mixtures between solvents), and contain various compounds such as waxes, fats, aromatic compounds (phenolics), terpenes, alcohols, esters, tannins, flavonoids, and carbohydrates [29]. Although extractives do not change the structure of wood, and they contribute only a low percentage of mass, they significantly impact its properties and transformation processes [30]. Recent reports on some Mexican hardwoods affirm that the content of extractive components ranges from 8.2 to 30.7% [19]. Our results show a percentage (11.37%) of extractives in *C. aesculifolia* within this range (Figure 2). We should mention that, according to the chemical and/or thermal transformation, the presence of extractives in lignocellulosic materials can have disadvantages, such as a greater risk of corrosion and an increase in the consumption of reagents in chemical transformation equipment. However, they may offer an advantage by increasing the higher heating value [27].

### 2.3. Proximal Analysis and the Higher Heating Value 

Proximal analysis is widely used to evaluate biofuels because it provides initial information on the possible qualities of a biomass as a fuel source. This method involves measuring such parameters as the moisture, ash, fixed carbon, and volatile compounds present in biomass samples [31]. Table 1 shows the results. It should be clarified that, in the case of gravimetric analysis, moisture is omitted because the calculations are made on a dry weight basis, i.e., 100% should be considered in relation to the percentages of volatile materials, fixed carbon, and ash.

#### 2.3.1. Moisture Content 

As Table 1 shows, the moisture content detected in *C. aesculifolia* was 9.37%, a figure within the range mentioned by other authors [32]. The marked variability of 6–75% in the initial moisture levels of our biomass samples was due primarily to the fact that some samples were taken from recently processed wood, while others were drawn from material that had been exposed to the environment. It is important to mention that high moisture content in biomass causes a weight increase that generates higher transport costs and greater energy consumption during the chipping process and can affect the quality of the splinters obtained [23]. High percentages of moisture in samples also require implementing drying procedures, although these raise costs and prolong drying times before the material can be used as biofuel [33].

It is worth mentioning that we have observed that humidity varies slightly even on the same day at different times. For that reason, the chemical analysis was performed with anhydrous wood, and the humidity of 9.37% corresponds to the humidity of the sample in the proximal analyses determined by gravimetric methods. It should also be mentioned that the proximal analyses were carried out on the basis of anhydrous weight; that is, the humidity of the flour was adjusted in the calculations. Due to the two procedures—chemical and TGA (Section 2.6) methods—there are some differences in the results, such as the humidity of 9.37% and 6.65% respectively.

#### 2.3.2. Volatile Materials

The proportion of volatile materials in lignocellulosic biomass can vary from 76 to 86% by weight [34]. *C. aesculifolia* had a volatile material content within this range, at approximately 84% (Table 1). This volatile fraction was converted into gas [35], which was also within the reported limits of 82.6–84.9% for biomass collected in diverse regions of Mexico [18]. In analyses of volatile materials, a high content is an indicator that favors ignition even at relatively low temperatures, as various authors have noted [36]. However, high levels can also increase the speed of the combustion process, which may be disadvantageous in the case of solid biofuels [37], although they can be beneficial for biogas production [38]. As can be seen, *C. aesculifolia* is rich in volatiles, which, when thermally degraded, can be implemented for the generation of biogas such as hydrogen from a reforming of the so-called synthesis gas. This product would be a significant bioresource for such important purposes as the generation of electrical energy from the use of fuel cells (for example, solid oxides) [39].

#### 2.3.3. Fixed Carbon 

Fixed carbon is generated by the combustion of biomass through the oxidation of solid material, which lasts considerably longer than that of gases in fuels [34]. *C. aesculifolia* had a fixed carbon value of 14.33% (Table 1), placing it in the highest range according to the figures of 14.1–14.3% reported for diverse hardwood species [14]. This factor is deemed favorable for bioenergy processes [40], although it is important to note that low fixed carbon values are not considered to be disadvantageous and, in fact, can be appropriate for biogas and biodiesel production [41].

#### 2.3.4. Ash 

Ash includes chemically bonded, salt-based compounds. Lignocellulosic materials acquire ash during growth in their natural environment. Individual species may have distinct components present in variable amounts [42]. The ash content in lignocellulosic materials ranges from 0.94 to 17.89% [43]. For the biomass under study, we found a percentage of 1.84% (Table 1). It is important to point out that some of the compounds that make up this ash are volatilized and incorporated into the gaseous phase, but that the residual part can cause serious problems in equipment (for example, corrosion and obstruction of the gaseous flow in combustion boilers). According to the aforementioned range, the ash content of *C. aesculifolia* (1.84%) is low, leading us to believe that these would not be common problems. Although low, this level would impede the fabrication of products like pellets and briquettes, since current norms stipulate a maximum ash content of 0.70 for the former and 0.5% for the latter [44]. 

#### 2.3.5. Higher Heating Value (HHV)

Another fundamental characteristic of biofuels is the higher heating value, which is determined by their chemical composition. It is widely held that elevated HHV values indicate good capacity as a fuel, while low values suggest the opposite [45]. Some researchers hold that the gross HHV of diverse raw materials ranges from 19.8 to 20.7 MJ/kg [34]. As Table 1 shows, the HHV value for *C. aesculifolia* wood (19.12 MJ/kg) is within this range. Similar reports exist for some hardwood species with high commercial value, such as the leaves of Ebenopis ebano [27]. This HHV result can be considered appropriate for the generation of high-value-added bioenergy products such as methane and hydrogen. This affirmation is based on the results observed in research on lignocellulosic biomasses that present an approximate HHV value of 19.12 MJ/kg, where gases such as bio-methane [46] and hydrogen [47] have been obtained from pyrolysis. However, due to the amount of fixed carbon obtained (14.33%), it is possible not to consider the production of materials such as briquettes, although this value may also represent an advantage in reducing CO_2_ emissions [48].

### 2.4. Microanalysis of Ash 

The results of the microanalysis of ash (see Table 2) identified 18 elements, with a marked presence of potassium (K), followed by calcium (Ca), phosphorus (P), sodium (Na), and magnesium (Mg). 

Of these five elements, the most common one (K) is characteristic of diverse woods and their derivatives, including Persian lime (leaves), orange trees’ branches, and lemon peel. In the case of the leaves of Persian lime, phosphorus and magnesium have similar values, and the value for sodium approaches those obtained for *Pinus* spp. [18]. The predominant presence of these elements is significant because they have the potential to cause complications during combustion processes by affecting the fusion point of ash, causing the formation of slag, corroding equipment, emitting fine particles, and causing incrustations that can damage ovens and boilers [49]. However, some authors stress that elements like Ca and Mg may favor combustion processes by raising the fusion point of ash, thus reducing the amount inside the combustion equipment and contributing to environmental safety by reducing the toxicity of the residues dispersed Into the air [50]. In contrast to these two elements, sodium, iron, and silicon interfere in the fusion of ash and lead to the formation of incrustations. Here, we should mention that sulfur (≈1200 ppm) can cause serious corrosion problems due to the formation of iron (II) chloride [46]. *C. aesculifolia* also had trace amounts of Cu, Ni, and Zn, which, even in small quantities, can have a direct negative impact on the environment. Actions have been undertaken to apply them to the benefit of the environment [51].

### 2.5. Elemental Analysis 

This analysis was utilized primarily to determine five elements: carbon, hydrogen, oxygen, nitrogen, and sulfur. The first three are important because they supply the highest amounts of energy in the biomass. However, nitrogen and sulfur, even in small amounts, are also very significant, for when derived from processes like pyrolysis they indicate the possible formation of gases and/or acids (Nox, Sox, NH_3_) that are harmful to the environment when expelled into the atmosphere. Table 3 shows the results of this analysis, which are within the ranges cited in recent publications. Of these five elements, we would emphasize the low sulfur content of *C. aesculifolia* (a value of just 0.08), although the nitrogen content is high. In thermal processes like pyrolysis, the atmospheric emissions of Sox types and nitrogenous compounds must be reduced to a minimum [52].

### 2.6. Thermal Properties of C. aesculifolia: TGA-DTG

**Figure 3:**
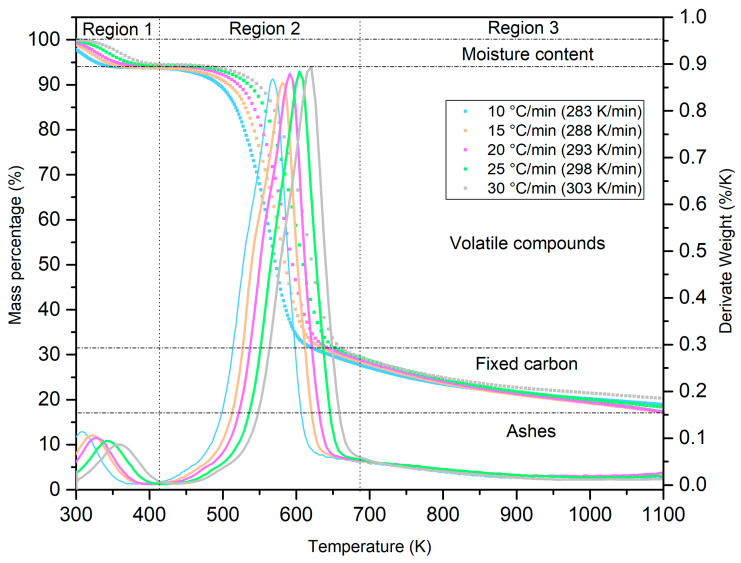
TGA-DTG test of *C. aesculifolia* for five heating rates.

The thermal behavior generated by the mass degradation of the lignocellulosic material under study was characterized using a thermogravimetric analyzer. In Figure 3, the two vertical axes show the loss of mass (left axis) at a certain velocity (right axis). The latter represents the first derivative (DTG) of the thermogravimetric curve (TGA) throughout the temperature range. The thermogram reveals a behavior similar to that of diverse lignocellulosic materials [54]. 

Three regions at distinct heating speeds are discernible in Figure 3 (β = 10–30 °C/min; 283–303 K/min). The first, located at temperatures between 300 and 415 K, can be identified with the loss of surface water from the biomass. As the graph advances, it presents the greatest loss of mass (≈60 %) in region 2 (415–680 K). For lignocellulosic materials, this zone is distinguished by the elimination of volatile materials, especially the two main polysaccharides: hemicellulose and cellulose. This is normally the area where the chemical reactions that decompose these compounds and the greatest generation of gases (methane, hydrogen, nitrogen, carbon monoxide and dioxide) occur, so it is called the active stage of pyrolysis. Finally, in the range of 680–1100 K, the degradation of lignin—the main cementing compound in these materials—occurs. Region 3 also reveals the formation of fixed carbon. In this stage, both curves (TGA and DTG) are practically flat.

In addition to the mass degradation curves (TGA), Figure 3 displays the characteristic peaks of the derivative of TGA—that is, the DTG that represents the ratio of change in weight loss with respect to temperature throughout the thermal process. Like TGA, DTG provides valuable information on key changes in the biomass, such as the maximum reaction temperatures (peaks) and decomposition velocities at any given point [55]. 

Parallel to TGA, the two main peaks in DTG represent the decomposition velocities of water, polysaccharides (hemicellulose and cellulose), and lignin. It is interesting to note that although this biomass is made up of these three compounds, as well as water, only two principal peaks encompass them. As mentioned, the polysaccharides degrade at distinct temperatures. Reports generally indicate that, due to heterogeneity and the size of the fiber in hardwoods [56], the decomposition of hemicellulose and cellulose occurs between 573 and 673 K [57], while the range for lignin is wider: 300–1200 K [58]. In this study, this summarizes the thermal behavior of *C. aesculifolia* wood. Another aspect observable in Figure 3 is the left-to-right shift of the TGA-DTG curves as the heating rate (10–30 °C/min; 283–303 K/min) and temperature increase. The explanation of this phenomenon is based on heat transfer inside the biomass, which is inversely proportional to the heating rate [59]. 

### 2.7. Kinetic Analysis of C. aesculifolia Wood: Ea and A

**Figure 4:**
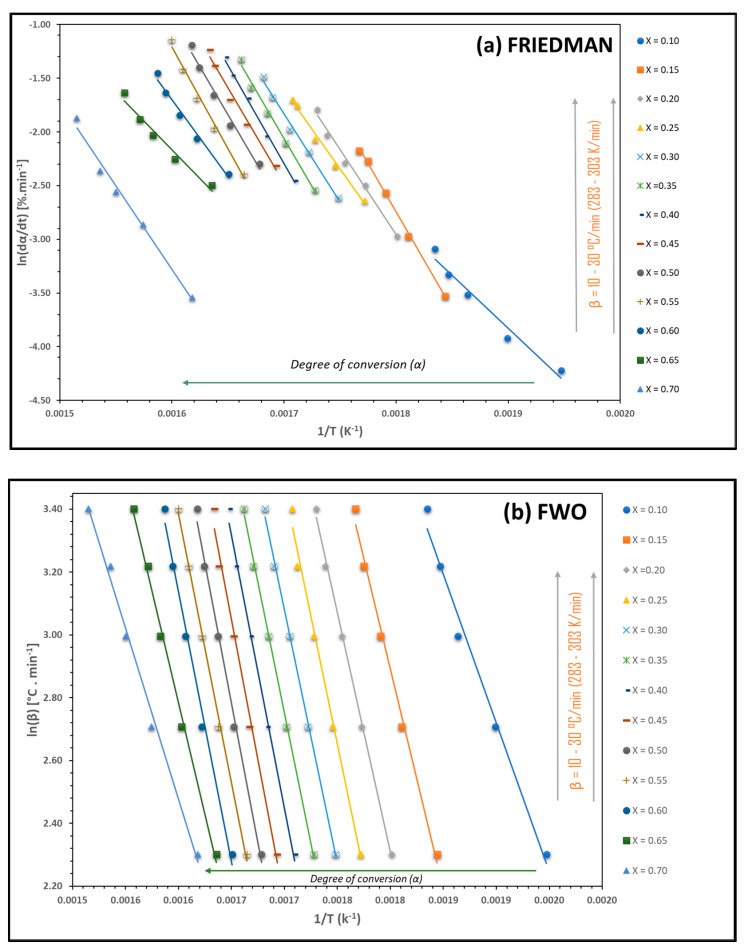

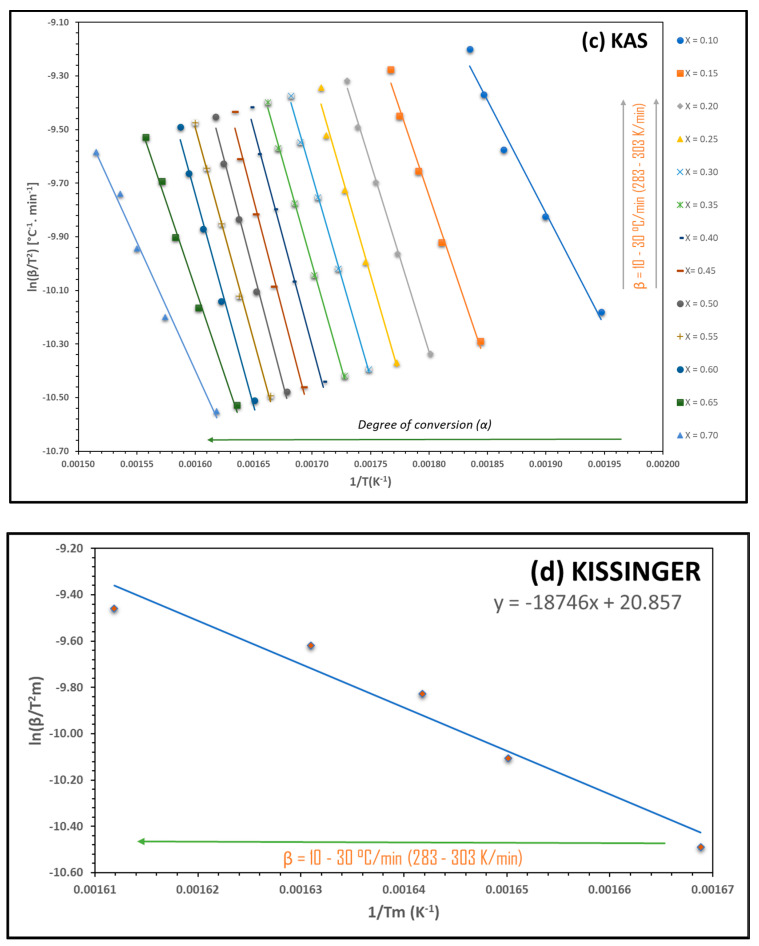
Regression lines to apparent activation energy proposed by Friedman (**a**), Flynn–Wall–Ozawa (FWO) (**b**), Kissinger–Akahira–Sunose (KAS) (**c**), and Kissinger (**d**) method free plots at the different heating rates for *C. aesculifolia* biomass.

Based on the data obtained from the thermogravimetric analysis and the methods of chemical kinetics proposed by Friedman, Flynn–Wall–Ozawa, Kissinger–Akahira–Sunose, and Kissinger, it was possible to determine the principal kinetic parameters—that is, activation energy (Ea) and the pre-exponential factor (A). The reaction order, meanwhile, was determined using Avrami’s method, as explained in the materials and methods section. These methods are extremely important for improving our understanding of the pyrolytic process of *C. aesculifolia* wood in an inert atmosphere. Since each one uses distinct aspects to determine the reaction mechanism, associated kinetics, and regression techniques according to the handling of the data obtained from the thermal process, distinct interpretations may be reached for each result, as occurs with Ea. Because each mathematical method applied in reaction kinetics studies represents diverse mechanisms, the values for the kinetic parameters (Ea and A) will show certain differences [60]. Figure 4a–d and Table 4 show the results for these parameters according to the four methods used to describe interrelations among the degree of advance, temperature, and velocity. They also show the calculation of the correlation coefficient, R^2^ (Table 4). This is particularly important because, according to the value obtained—preferably between 0.90 and 1.0—any graph of the kinetic methods proposed is considered acceptable. In this study, the graph of *C. aesculifolia* presented low R^2^ values (<0.80) when the degree of conversion (α) was ˃0.70. These values were not included in the analysis. It is important to mention that some research related to kinetic studies (Friedman, FWO, and KAS) of biomass have reported similar behavior, in fact with a lower degree of progress (α = 0.65). This situation can be attributed to the degree of complexity in the generation of carbon in the pyrolysis process [61]. Recent research has had to adjust its results to values where the degree of advance is also 0.70 due to poor fitting of the data—for example, in Trapa natans peel biomass [62]. However, this does not mean that the results are not valid for the study in question.

It is important to emphasize that the average R^2^ value for the four methods was ˃0.95, a figure practically equal to those of the FWO (0.9938) and KAS (0.9865) methods. We should clarify that Kissinger’s method does not use the degree of conversion as its basis for analysis, although it must also fulfill the condition that R^2^ is ˃0.90 in order to achieve more approximate values [63]. According to Table 4, the average Ea of the Friedman, FWO, and KAS methods was 132.03, 121.65, and 118.14 kJ/mol, respectively, while the figure for Kissinger’s method was 155.85 kJ/mol. The first three results are quite similar, with KAS having the lowest value and Kissinger’s method the highest. These differences are due to the mathematical bases employed in each method. Friedman’s method is differential, based on the rate of change in the degree of advance with respect to time (dα/dt), while FWO and KAS are based on the heating rate (β), and both are integral methods. Thus, we must keep in mind that these kinetic methods are complementary and should not be understood as being in competition [64]. Also important, however, is that Kissinger’s method reports only a value for Ea. Since it is not possible to appreciate the degree of advance of the pyrolytic process, the complexity of that process is not observed clearly. From the phenomenological point of view, one of the variables used by these methods (Friedman, FWO, KAS, and Kissinger) is temperature. Such differences in activation energy can also be explained as a function of increasing temperature and advancing degree of advancement, causing a reduction in the molecular mobility of the biomass structure, resulting in an increase in activation energy. As the process continues, the activation energy is reduced as the kinetics progresses [65]. It can be observed that the value of the activation energy in the FWO method is slightly higher compared to the KAS method; this may be related to the behavior of the bonds in the biomass structure, the weakest being the ones that are first randomly broken by scission effects [66].

Table 4 also shows that the maximum values for Ea (Friedman, KAS, FWO) are within the range of the degree of advance (α)—that is, between 0.40 and 0.55. This means that, based on this value (0.55), the amount of mass available to participate in the thermal process will be lower. These maximum values can also be interpreted as a function of the greater decomposition of the biomass that, according to the TGA-DTG graph (Figure 3), corresponds to the elimination of the HM and CE that make up the wood of *C. aesculifolia*. 

Another significant aspect is the variability in Ea over the range considered for the methods applied: 81.89–153.10, 74.79–141.78, and 69.89–138.95 kJ/mol for the Friedman, FWO, and KAS methods, respectively. These results mean that the pyrolytic process is complex, occurs in multiple stages, and presents diverse types of chemical reactions across the temperature range [67]. In general, reports in the literature affirm that the range of Ea for lignocellulosic biomass made up of woody tissue lies between 60 and 170 kJ/mol [68]. Our results are within this range. Other biomasses present similar values for Ea: nutshell (≈136 kJ/mol) [69], Indian almond (≈133 kJ/mol) [70], Brazil nut (≈137 kJ/mol) [71], products of figs (≈160 kJ/mol) [72], fistula cane (Cassia fistula L.) (≈137 kJ/mol), peach palm (≈112 kJ/mol) [73], Manilkara zapota seeds (≈132 kJ/mol), Delonix regia (≈143 kJ/mol), and Cascabela thevetia (≈152 kJ/mol) [74]. 

**Figure 5:**
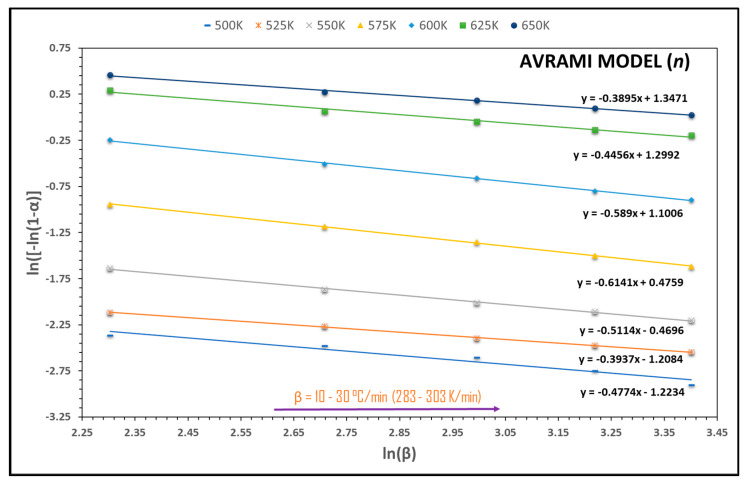
Regression lines to reaction order (*n*) proposed by Avrami’s theory.

Another key parameter in kinetic analyses of thermogravimetric processes is the reaction order (*n*). The variation in this parameter for *C. aesculifolia* is shown in Figure 5 and Table 5. This was calculated using Avrami’s equation, as indicated in the Materials and Methods section. Calculating the reaction order requires considering the heating rate (β = 10–30 °C/min; 283–303 K/min) and the temperature range of 500–650 K for the most important event of pyrolysis (region 2)—that is, the TGA-DTG analysis. Clearly, according to the lines in Figure 5, the value of the lineal correlation coefficient (R^2^) lies between 0.95 and 0.99, with an average of 0.9903, which is acceptable for calculating the reaction order.

As Table 5 shows, the reaction order initially increased from 0.3937 to 0.6141, before decreasing to 0.3895, with an average value of 0.4887. This result is similar to those obtained by authors who have analyzed pyrolysis using agricultural waste and applying Avrami’s equation, as in the cases of corn straw and rice husk, for which average reaction orders of 0.365 and 0.539 were reported, respectively. It is true that this value can change from one type of biomass to another. These differences may be related to the composition of ash content, since this exerts a significant effect on the alkaline metals present and, in turn, their impact on the thermal process [75]. 

### 2.8. Thermodynamic Analysis of C. aesculifolia: A, ΔH, ΔG, and ΔS

The first parameter obtained in thermodynamic analyses is the pre-exponential factor, calculated using the Ea values throughout the range of the degree of advance (α = 0.10–0.70) and the temperature at the maximum peak of the DTG (Figure 3). Table 4 shows the pre-exponential factor (A) obtained at a low heating rate (β = 15 °C/min). This reflects the fact that the frequency of molecular collisions increases proportionally to the heating rate [76]. In this regard, applying a low value of β reduces the degree of interaction among the constituents of the biomass in pyrolysis. Recent research (on cattle manure and pistachio shells) has also opted for the calculation of the pre-exponential factor at even lower heating rates (10 °C/min) [77,78]. It is worth mentioning that, with respect to different regions of mass degradation, more accurate results can be obtained by performing thermogravimetric analysis at low heating rates, even as low as 1 °C/min [79]. In this regard, and based on the information reported in the aforementioned investigations, thermodynamic analysis of *C. aesculifolia* is performed at a low heating rate. In this study, *A* varied in the range of 10^4^–10^11^ s^−1^, defining the occurrence of both reactions and complex structures in this process due to the thermal conversional of the biomass [60]. This variability in *A* may be conditioned by other factors, such as particle size, the presence of a catalyzer, and even the diverse heating rate applied. It is indicative of the collisions that occur among the particles during the thermal process. Here, the number of collisions increased with the increase in this value. The average values shown in Table 4 for the KAS, FWO, Friedman, and Kissinger methods are 2.41E + 09, 4.30E + 09, 8.11E + 10, and 3.47E + 11 s^−1^, respectively. 

Similar results have been reported where A varied from 10^7^ to 10^12^ s^−1^—for example, with red pepper, rice husk, and bran [80]. Other reports affirm that, depending on whether the value of A is <10^9^ s^−1^ or ≥10^9^ s^−1^, either collisions or superficial reactions and the formation of a simple compound will occur, respectively. However, if this value lies between 10^10^ and 10^12^ s^−1^, there is a possibility of activating rotations of certain compounds that were passive at first [81]. This may cause the size of the complex formed to increase (unimolecular reaction) or remain unchanged (monomolecular reaction) in relation to the interaction with its neighbors [82]. In this case, regardless of the method applied, the constant values of the pre-exponential factor (10^10^–10^11^ s^−1^) represent the zone where the main loss of mass occurs—that is, degradation of HM and CE when the degree of advance (α) lies between 0.40 and 0.55 throughout the pyrolytic process. The highest value for *A* (3.47E + 11 s^−1^) was calculated by Kissinger’s method, perhaps reflecting the fact that the rotation of the active–reactive compound pair remained unchanged during the thermal process [83]. However, in light of the disparity with the results of the Friedman, FWO, and KAS methods, it is likely that Kissinger’s method presented this result because of the method’s mathematical basis, as explained previously. 

In addition to the pre-exponential factor, and as part of the thermodynamic study of the pyrolysis process of *C. aesculifolia*, Table 6 shows the variation in three other key parameters that make it possible to analyze energy behavior in terms of spontaneity, conservation, equilibrium, and quality [84], namely, analyses of enthalpy (ΔH, kJ/mol), Gibbs free energy (ΔG, kJ/mol), and entropy (ΔS, J/mol. K). As mentioned above, these parameters were calculated at a low heating rate. Table 6 displays the average values for these thermodynamic properties (ΔH, ΔG, and ΔS) according to the Friedman (127.12, 174.89, −78.83), FWO (116.75, 175.30, −96.61), KAS (113.23, 175.46, −102.68), and Kissinger (150.95, 173.97, −37.98) kinetic methods.

In the process of lignocellulosic biomass pyrolysis, i.e., the generation of compounds such as solids, liquids, and gases, the enthalpy can be defined as the energy that will be required by the biomass for the generation of these products [85]. Table 6 shows that, regardless of the mathematical method applied, enthalpy begins with low values, increases proportionally with the reaction order up to high values, and then decreases. This behavior revealed that the biomass contained compounds that require greater energy to achieve their total transformation. The positive value of enthalpy is another important factor, for it indicates that the thermal degradation process of *C. aesculifolia* involves an endothermic reaction [86]. Recently, similar enthalpy values have been obtained from the biomass of Manilkara zapota seeds, with reports of an average value of 137 kJ/mol [75]. Other studies, one with almond husk and another with acorn pericarp [87], have reported values close to the ones found in our work. Here, we would emphasize that the difference between activation energy (Table 4) and enthalpy (Table 6) is <5 kJ/mol, so we can affirm that the results for the degradation of *C. aesculifolia*, the formation of activated compounds, and the conversion into other compounds will all be favorable [88]. This means that the energy barrier to carry out the pyrolysis process is low, and the formation of pyrolytic products will be favorable.

In lignocellulosic biomass pyrolysis processes, the possibility of generating activated complexes is defined according to the total energy available in the thermal system, i.e., Gibbs free energy (ΔG) [82]. As Table 6 shows, this parameter is positive throughout the range of the degree of advance (α = 0.10–0.70), indicating that the process does not develop directly or automatically but, rather, is oxidative in nature and requires external energy to achieve the pyrolysis reaction [89]. This need for additional energy input may be a disadvantage; however, it is important to highlight that, in pyrolytic biomass processes, the results of entropy (negative) and ΔG > ΔH (Table 6) imply consideration that a small amount of energy is surplus as input to the thermal system [90]. It is also clear that an approximate value of 175 kJ/mol is maintained in all three iso-conversional methods (Friedman, FWO, KAS), as well as for Kissinger’s non-iso-conversional method. Recent studies of corncobs and pine wood report approximate ΔG values of 173 kJ/mol [91] and 180 kJ/mol [86], respectively. These values are relatively high for lignocellulosic biomass, which means that pyrolysis of *C. aesculifolia* consumes a large amount of energy. 

Theoretically, the entropy (ΔS) of a system represents its molecular disorder and randomness [76]; however, in a pyrolytic system, this function may represent the level of order or disorder of the carbon layers formed in the thermal process [82]. Results for this factor are shown in Table 6, where negative values indicate that the system will undergo only relatively small physicochemical changes; that is, the level of disorder in the thermal process of *C. aesculifolia* is relatively low compared to some of its degraded products [92]. The highest ΔS value was found for the KAS method (−102.68 J/mol. K), while the lowest was found for Kissinger’s method (−37.98 J/mol. K). Studies of biomass waste (red pepper) have reported such negative values ranging from −100 J/mol. K to almost −250 J/mol. K over a wide conversion range. [93]. This variability in entropy may be related to the mathematical method applied and to the precise components of the biomass, which can interfere with the thermal process, such as the presence of certain inorganic and alkaline earth compounds, or even the production method of the biomass [94]. However, it has also been determined that such negative entropy values (i.e., reduction in the randomness of the system) as those reported in this study for *C. aesculifolia* can be generated due to physicochemical aging processes, which can lead to a higher thermodynamic equilibrium [95].

### 2.9. Fourier-Transform Infrared Analysis (FT-IR)

**Figure 6:**
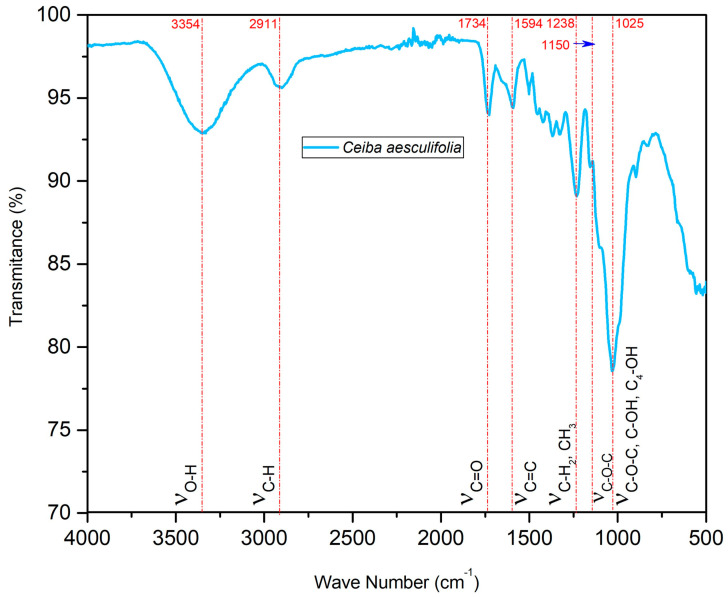
FT-IR spectra of *C. aesculifolia*.

The FT-IR spectra of raw *C. aesculifolia* biomass are portrayed in Figure 6. There, we can identify the characteristic signals that correspond to the various functional groups that constitute the structure of wood, including polysaccharides, lignin, and other low-weight molecular compounds. The absorption band visible at 3354 cm^−1^ corresponds to the stretching vibrations of hydroxyl groups (OH) that are interlaced intermolecularly. These vibrations are related to the main constituents of wood, including cellulose, hemicellulose, lignin, and some proteins [75]. In another study, this functional group was reported for the bark of *C. pentandra* at 3418 cm^−1^ [96]. There are reports in the literature that this functional group is associated with the constitutional water present in the cell walls of wood [97]. Another functional group identified was CH, as has occurred with other species of this genus, like the bark of C. pentandra, where a similar signal to that of *C. aesculifolia* was observed at 2935 cm^−1^ [96]. According to the literature, the signal at 2911 cm^−1^ is associated with symmetric and non-symmetric vibrations, suggesting the presence of groups of aliphatic chains (CH_2_, CH_3_) derived from the elemental structure of the raw material, such as cellulose, hemicellulose, and lignin [98]. The CH functional group has been found in other studies of hardwood species in the range of 2938–2933 cm^−1^ [99]. Likewise, for species of this genus, there are reports of a vibration in the region around 2918 cm^−1^ in the cellulose of the fruit of *C. pentandra* [100]. Published reports also affirm that the bands centered around 2911 cm^−1^ have been associated with extractable substances located on the surface of the primary cell wall. A study of *C. aesculifolia* seeds found these kinds of substances at 2918 cm^−1^, while another found them in C. speciosa fruit (also a member of this genus). In that case, the signals at 2900 cm^−1^ were attributed to lipids [101,102]. 

The signal at 1734 cm^−1^ is associated with stretching vibrations of the C=O type, such as the aldehyde, ketone, and ester functional groups present in cellulose, hemicellulose, and lignin [103]. This finding is similar to those of a study on *C. aesculifolia* seeds, which reported a signal at 1736 cm^−1^ [102], and to those of reports on hardwoods that found this functional group between 1740 and 1730 cm^−1^ [104]. In the absorption band centered at 1594 cm^−1^, there were stretching vibrations characteristic of double bonds (C=C) in the aromatic skeleton of lignin [105]. A study of *C. aesculifolia* seeds reported this group (C=C) at 1594 cm^−1^, a result consistent with the value reported here [102]. The functional groups CH_2_ and CH_3_ have been found in studies of hardwood species at around 1464–1375 cm^−1^ [104]. The signal at 1238 cm^−1^ is also linked to those groups, associated mainly with the CE and lignin in *C. aesculifolia* [106]. This signal has also been associated with the stretching of the C-O bonds in the xylene and syringyl ring of lignin and hemicellulose [107]. A study of cellulose using *C. pentandra* fruit identified C-O at 1249 cm^−1^ [99]. 

The peaks at 1150 cm^−1^ are assigned to the stretching vibration of the C-O-C bridge of the characteristic esters of cellulose and hemicellulose. One study of *C. aesculifolia* seeds reported this bond at 1157 cm^−1^ [100]. The functional groups C-O-C, C-OH, and C_4_-OH were identified in the absorption band at 1025 cm^−1^, where mainly β-glucopyranose was located in the cellulose [101]. Similar values have been obtained for other hardwood species, including Prosopis laevigata [101]. Some authors affirm that the signal centered at 1025 cm^−1^ corresponds to the symmetric stretching of the C-OH groups in lignin, cellulose, and hemicellulose [108].

There are reports that vibrations around 930 cm^−1^ correspond to glycosidic bonds in cellulose and hemicellulose [109]. A study of cellulose in *C. pentandra* fruit identified this at 900 cm^−1^ [100]. Likewise, the vibration around 725 cm^−1^ can be attributed to the balance of the CH_2_ group in crystalline cellulose I [110]. A study of *C. aesculifolia* seeds located this at 710 cm^−1^ [102]. These peaks are characteristic of native cotton [111]. Finally, the signal found with values near 600 cm^−1^ was thought to be due to the deformation of OH groups [112]. A study of cellulose in *C. pentandra* fruit identified this at 614 cm^−1^ [100].

## 3. Materials and Methods

### 3.1. Collection Area and Preparation of the Study Materials 

**Figure 7:**
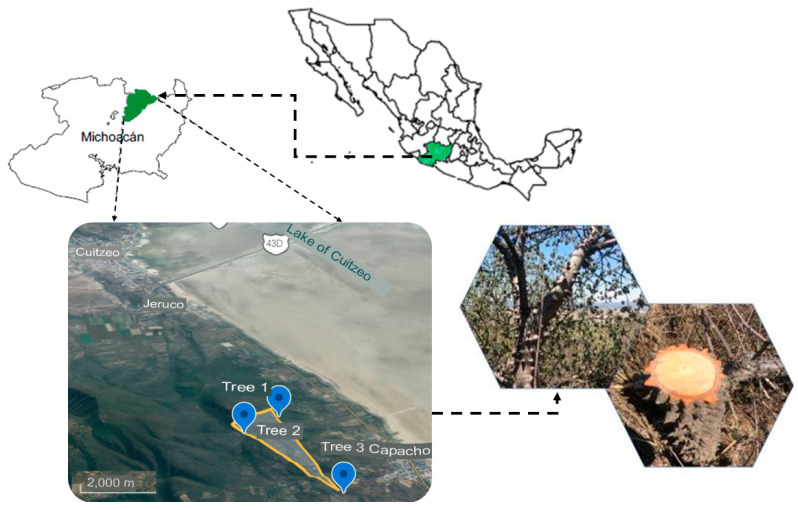
Geographic location of *C. aesculifolia*.

As Figure 7 shows, the *C. aesculifolia* waste analyzed was collected in the Lake Cuitzeo basin in the state of Michoacán, Mexico (coordinates in Table 7). Three trees were selected, and 10 cm wide slices were cut at a height of 1.30 m (DAP: diameter at the height of the chest). We then separated the wood and bark of the slices. The wood was splintered manually and left to dry in the shade until it reached moisture equilibrium (approximately 12%). After drying, the splinters were pulverized in a mill (Model K20F, series 236, Micron S.A. de C.V., Mexico City). Finally, the pulverized material was sifted in a Ro-Tap machine (Model RX-29, W.S. Tyler, Mentor, OH, USA), utilizing meshes of 20, 40, and 60. The material from flour mesh 40 (425 µm) was used for the chemical, energy, and thermal characterizations (TGA-DTG) and thermodynamic analysis.

### 3.2. Determination of Basic Density 

Three pieces of *C. aesculifolia* wood were selected from each tree, following the recommendation to take samples that are free of knots and in good condition with a moisture content of 12%. The samples were cut into rectangles to facilitate measuring. A Vernier caliper was used to precisely measure the length, width, and thickness of each sample in order to calculate their volume [113]. Mass was calculated on an Ohaus PA214 scale at a precision of 0.0001 g, and density was determined (g/cm^3^).

### 3.3. Basic Chemical Composition 

The chemical composition was verified according to well-known methodologies [114]. The main polymeric components of the wood (CE, HM, lignin) were determined by fiber analysis using Van Soest’s gravimetric method with α-amylase. Extractable substances were calculated by subtracting the percentages of CE, HM, lignin, and ash at 100% application, as well as a correction factor for ash as in earlier studies [115]. The equipment utilized was an ANKOM fiber analyzer (model AMKON200, ANKOM Technology, Macedon, NY, USA).

### 3.4. Proximal Analysis 

The moisture content of each dry sample was determined in triplicate based on the UNE-EN ISO18134-1 standard [116]. The percentage of ash was calculated according to the UNE-EN 14775 standard [117], and the volatile material content was determined in accordance with ASTM E 872-82 [118]. As Equation (1) shows, fixed carbon [119] was calculated by the differences of the means. Average values and standard deviations are reported.

(1) %Fixed carbon=100−(%volatile matter+%ash)

### 3.5. Microanalysis of Ash

This analysis was conducted by inductively coupled plasma optical emission spectrophotometry (VARIAN 730-ES, Varian Inc., (Agilent), Mulgrave, Australia), following well-established procedures [120].

### 3.6. Elemental Analysis 

This analysis encompassed determining the primary components of the biomass, such as carbon, hydrogen, oxygen, nitrogen, and sulfur (CHONS). Calculations of C, H, N, and S were carried out in triplicate using a COSTECH elemental analyzer (Model 4010; COSTECH International S.P.A., Milan, Italy), in strict adherence to the UNE-CEN/TS 15104 EX standard [121]. Oxygen content was calculated from the difference in each analysis performed.

### 3.7. Higher Heating Value (HHV)

The higher heating value was determined in triplicate according to the UNE-EN 14918 standard [122], using a semiautomatic calorimeter (LECO AC600, LECCO Corporation, St. Joseph, MO, USA). The calorimetric pump was calibrated with benzoic acid. Mean values and standard deviations are reported.

### 3.8. Fuel Value Index

It is necessary to evaluate lignocellulosic material destined for use as fuel in terms of its suitability for bioenergy production. In this regard, it is important to mention that the fuel value index is a measure used to categorize the biomass by taking its heating value and density as positive parameters, and ash as a negative one. This analysis was performed in triplicate. Average values and standard deviations are reported. This parameter was also determined using well-established methods according to Equation (2) [123].

(2) $$Fuel\;value\;index=\frac{\left(Higher\;heating\;value,\;MJ/kg\right)\left(basic\;density,\;g/{cm}^{3}\right)}{ash\;content\;(\%)}$$

### 3.9. Thermogravimetric (TGA-DTG), Thermodynamic, and FT-IR Analyses

#### 3.9.1. TGA-DTG Analysis

**Figure 8:**
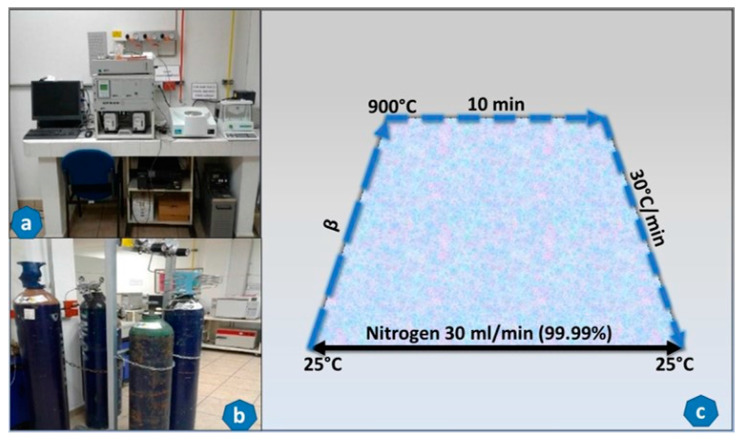
Equipment for thermogravimetric analysis (**a**), nitrogen tanks used in the experiment (**b**) and heating ramp used (**c**).

The thermal analysis (TGA-DTG) of *C. aesculifolia* wood (Kunth) Britt and Baker was carried out with an STA 6000 device (Perkin Elmer Inc., Wellesley, MA, USA). Pyrolytic conditions were maintained using ultrahigh-purity nitrogen gas (99.99% pure) acquired from INFRA, Mexico. This grade of purity was required to minimize possible mass transfer. The nitrogen flow was kept at 30 mL/min. Approximately 30 ± 5 mg of the sample was placed uniformly in an aluminum crucible for each assay, heated through a programmed range of 25–900 °C, and kept at this temperature for 10 min, before being allowed to cool at a rate of 30 °C/min. Figure 8 depicts this process. It is important to note that the kinetic and thermodynamic analyses of *C. aesculifolia* waste required considering as many as five heating rates (β = 10–30 °C/min; 283–303 K/min). To minimize error and achieve greater precision, all tests were performed in triplicate. The heating ramps were also carried out in triplicate when the conversional difference was ˃5%, or when the data contained excessive background noise. OriginPro Graphing and Analysis software 2016 was used to graph the data.

#### 3.9.2. Theory of Kinetic Analysis 

**Figure 9:**
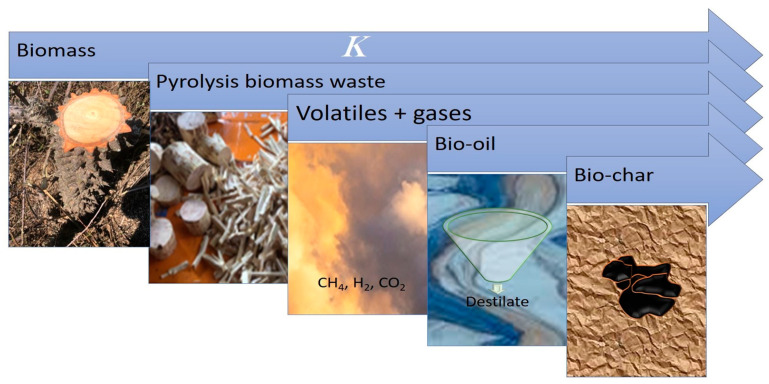
General biomass pyrolysis process.

According to the iso-conversion principle, the methods used for kinetic analyses are based on a constant conversional value, such that the reaction velocity is a function of temperature [121,122]. The pyrolysis process of solid biomass can be simplified as a one-step global process with a first-order reaction, as depicted in Figure 9. 

According to Figure 9, volatiles are considered to be indicative of the gas generated, while the variable *k* refers to the velocity constant of the pyrolysis process. The conversion degree in each fraction, denoted as *α*, in any temperature range, is defined as the mass conversion in the pyrolytic reaction. It can be defined as in Equation (3):

(3) $$\alpha =(m_{i}-m_{t})/(m_{i}-m_{f})$$

where *m_i_*, *m_t_*, and *m_f_* represent the initial, instant, and final mass of the sample, respectively. 

Parallel to this, the constant of velocity (*k*) is directly related to the temperature (*T*) of the entire pyrolytic process. As Equation (4) shows, Arrhenius’s ratio makes it possible to analyze this relation:

(4) $$k(T)=A{exp}^{\left(\frac{-E_{a}}{RT}\right)}$$

This equation includes several variables of vital importance for analyses of thermogravimetric processes. The indicators *A*, *Ea*, *R*, and *T* represent the pre-exponential factor (s^−1^), the apparent activation energy (kJ/mol), the universal constant of ideal gases (8.314 J/Kmol), and the absolute temperature (Kelvin), respectively.

Equation (4) can be considered together with a reaction method (*fα*) in order to determine the ratio of change in the degree of mass conversion with respect to time (*dα/dt*), as shown here:

(5) $$\frac{d\alpha }{dt}=K\left(T\right)\times f(\alpha )$$

By substituting the value of *k(T)* from Equation (4) into Equation (5), we obtained the fundamental expression of the analytical methods applied to study the kinetic parameters in relation to the results of the thermogravimetric analysis:

(6) $$\frac{d\alpha }{dt}=A{exp}^{\left(\frac{-E_{A}}{RT}\right)\times f(\alpha )}$$

Non-isothermal processes generally consider a heating rate (*dT/dt*), denoted as β. Upon including this variable in Equation (6) and simplifying it, we obtained

(7) $$\frac{d\alpha }{dt}=\frac{A}{\beta }\cdot {exp}^{\left(\frac{-E_{A}}{RT}\right)\times f(\alpha )}$$

The solution of this expression can be approached using methods like those of Doyle [123], Agrawal, Gorbatchev, and Frank-Kamenetskii [124], which permit calculating the principal kinetic parameters involved in the thermal process. The resulting equation is

(8) $$g\left(\alpha \right)=\int_{0}^{\alpha }\frac{d\alpha }{f(\alpha )}=\frac{A}{\beta }\int_{T_{0}}^{T}{exp}^{\left(\frac{-E_{a}}{RT}\right)dT}$$

##### Determination of the Kinetic Parameters

**Figure 10:**
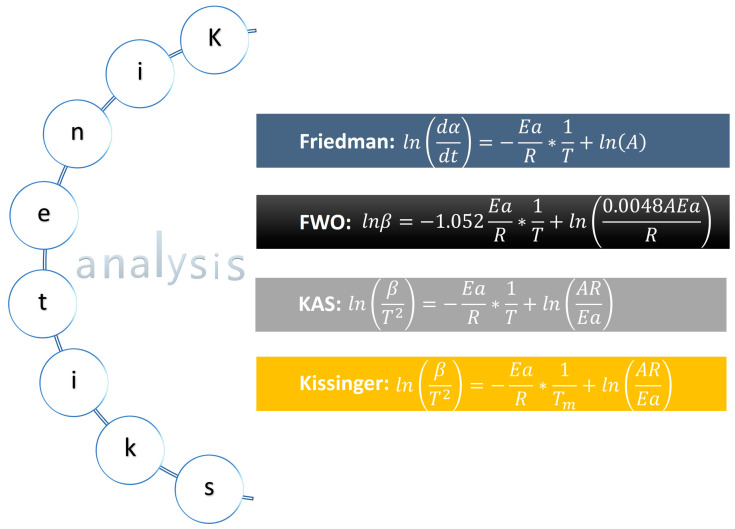
Mathematical method to determine the kinetic parameters of *C. aesculifolia*.

Since a complex physicochemical interaction of mass–heat transfer occurs during pyrolysis, it is necessary to optimize the energy and/or reduce contaminants in the industrial equipment used in this process of thermally transforming lignocellulosic material like the biomass analyzed. This means that a study of the kinetics involved must be conducted first to analyze the main parameters: activation energy, the pre-exponential factor, and the reaction order. Figure 10 shows some of the mathematical methods most often employed to calculate the first two parameters—the same ones that were applied in this study.

To calculate the reaction order, we began with Avrami’s equation [124]. The ratio required for this parameter is outlined in the following procedure:
Avrami’s method:$\alpha =1-e^{\left(\frac{-k\left(T\right)}{\beta^{n}}\right)}$Transposing and applying logarithms:$-ln\left(1-\alpha \right)=-\left[\left(\frac{-k\left(T\right)}{\beta^{n}}\right)\right]$
$-ln\left(1-\alpha \right)=-\left[\frac{-k\left(T\right)}{\beta^{n}}\right]$
$-ln\left(1-\alpha \right)=\left[\frac{k\left(T\right)}{\beta^{n}}\right]$Applying logarithms again:$ln\left[-ln\left(1-\alpha \right)\right]=ln\left(\frac{k\left(T\right)}{\beta^{n}}\right)$Applying logarithmic properties: $ln\left[-ln\left(1-\alpha \right)\right]=lnk\left(T\right)-ln\beta^{n}$Substituting *k*(*T*) according to Equation (3):$ln\left[-ln\left(1-\alpha \right)\right]=ln\left[Ae^{\left(-\frac{Ea}{RT}\right)}\right]-ln\beta^{n}$Applying logarithmic properties:$ln\left[-ln\left(1-\alpha \right)\right]=lnA+\left(-\frac{Ea}{RT}\right)-ln\beta^{n}$
$ln\left[-ln\left(1-\alpha \right)\right]=lnA-\frac{Ea}{RT}-ln\beta^{n}$Equation that calculates the reaction order:$ln\left[-ln\left(1-\alpha \right)\right]=lnA-\frac{Ea}{RT}-nln\beta$The graph is obtained in analogy to the equation y=mx+b, where$y=ln\left[-ln\left(1-\alpha \right)\right]$ m=−n x=lnβ $b=lnA-\frac{Ea}{RT}$

Hence, the value for the reaction order (*n*) is given by the slope (*m*) calculated on the *y* vs. *x* graph.

#### 3.9.3. Determination of the Thermodynamic Parameters 

**Figure 11:**
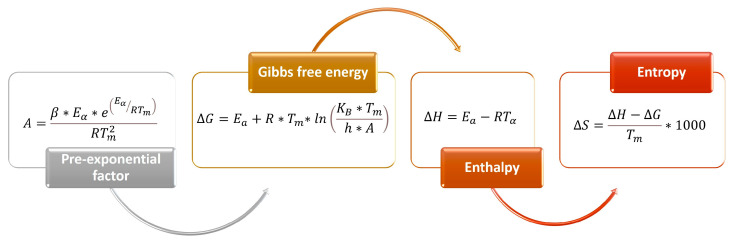
Equations for the determination of thermodynamic parameters, where *E_a_* is the activation energy, *R* is the ideal gas constant, *T_α_* is the temperature according to the degree of conversion, *T_m_* is the temperature at the maximum peak of the DTG, *K_B_* is the Boltzmann constant (1.381E-23 J/K), *h* is Planck’s constant (6.626E-34 J.s), and β is the heating rate (15 °C/min).

Once *Ea* was determined by the Friedman, FWO, KAS, and Kissinger methods, we were able to calculate the three thermodynamic parameters enthalpy (Δ*H*), Gibbs free energy (Δ*G*), and entropy (Δ*S*), at a low heating rate of 15 °C/min. In this stage, we also calculated the pre-exponential factor (*A*) considering, first, the *Ea* value obtained and, second, the heating rate. Figure 11 shows the equations utilized.

#### 3.9.4. Fourier-Transform Infrared Analysis (FT-IR)

The FT-IR spectrum was obtained from the raw biomass. The functional groups of *C. aesculifolia* waste were analyzed by FT-IR in a Perkin Elmer ATR model 400 spectrometer. Spectra were obtained in 16 scans per sample in a range of 4000–500 cm^−1^, at a resolution of 4 cm^−1^. The samples selected were mixed with potassium bromide (KBr) and then dried in an oven for 24 h.

## 4. Conclusions

The waste of *Ceiba aesculifolia* wood constitutes an abundant renewable energy source. This study was designed to determine the thermochemical characterization of this biomass during pyrolysis. The results for moisture (9.37%), ash (1.84%), volatile material content (83.83%), and HHV (19.12%) demonstrate that it is feasible to consider this biomass an excellent candidate with high energy potential. The TGA analysis indicated that the greatest degradation, around 60%, occurred in the temperature range of 415–680 K. This is the first study to conduct a kinetic analysis of the thermogravimetric process of *C. aesculifolia* waste with a view to determining the kinetic triplet using three iso-conversional methods and one non-iso-conversional approach. The average values estimated for activation energy by the Friedman, FWO, KAS, and Kissinger methods were 132.03, 121.65, 118.14, and 155.85 kJ/mol, respectively. The Friedman, FWO, and KAS methods showed variations throughout pyrolysis, but Kissinger’s method showed no changes. According to Avrami’s equation, the reaction order presented a variation of 0.3937–0.6141 across the entire temperature range (500–650 K). It is important to emphasize that a high correlation coefficient was obtained (R^2^ = approximately 1) in all of the graphs of the mathematical methods applied, a result that is deemed preferable in kinetic analyses. The value for the pre-exponential factor varied from 10^3^ to 10^11^ s^−1^, indicating that pyrolysis of *C. aesculifolia* waste transpires at a high reaction velocity. The thermodynamic analysis showed that the pyrolysis was endothermic, since the difference between the apparent activation energy and enthalpy was <5 kJ/mol. We conclude that this process is not totally spontaneous, since the Gibbs free energy and entropy were positive and negative, respectively. The principal vibrations detected by FT-IR were O-H, C-H, C=O, C=C, CH_2_, CH_3_, C-O-C, C-OH, and C_4_-OH. Based on these findings, we can affirm that it is possible to optimize the times, temperatures, and heating rates of *C. aesculifolia* waste during pyrolysis to generate bioenergy.

## Figures and Tables

**Table 1:** Average results of proximate analysis and superior calorific value of *C. aesculifolia*.

Moisture (%)	VM (%) ^1^	FC (%) ^2^	Ash (%)	HHV (MJ/kg) ^3^
9.37 ± 0.2	83.83 ± 2	14.33 ± 2.1	1.84 ± 0.1	19.12 ± 0.2

^1^ VM: volatile material, ^2^ FC: fixed carbon, and ^3^ HHV: higher heating value.

**Table 2:** Microanalysis of *C. aesculifolia* ashes.

Element	ppm
K	257,523.69
Ca	35,917.89
P	15,824.95
Na	2373.19
Mg	1329.53
S	1198.21
Sr	292.95
Si	164.05
Ba	153.00
Fe	112.42
Li	91.78
B	69.67
Cu	41.73
Al	38.88
Mn	35.36
Zn	26.46
Ni	0.90
Cr	˂0.05

Note: ppm = parts per million. The elements are represented by their symbols.

**Table 3:** Elemental analysis of *C. aesculifolia* wood.

Element	This Research (%)	Literature (%) [42,53]
C	45.60	40–46
H	6.90	4–6
O	47.20	45–50
N	0.30	0.11–0.32
S	0.08	0.00–0.96

**Table 4:** Kinetic parameters for pyrolysis of *C. aesculifolia* biomass.

Degree of Conversion (α)	Equation (*y* = *mx* + *b*)	*R* ^2^	*Ea* (kJ.mol^−1^)	*A* (s^−1^), (β = 15 °C/min)
**Friedman method**				
0.10	y = −9849.9x + 14.886	0.9675	81.89	7.68E + 04
0.15	y = −18044x + 29.732	0.9981	150.02	1.05E + 11
0.20	y = −15786x + 25.46	0.9892	131.24	2.21E + 09
0.25	y = −14761x + 23.488	0.9894	122.72	3.81E + 08
0.30	y = −16454x + 26.142	0.9919	136.80	6.93E + 09
0.35	y = −17888x + 28.348	0.9921	148.72	8.03E + 10
0.40	y = −18415x + 29.008	0.9946	153.10	1.97E + 11
0.45	y = −17748x + 27.696	0.9821	147.56	6.33E + 10
0.50	y = −17833x + 27.585	0.9812	148.26	7.31E + 10
0.55	y = −18989x + 29.174	0.9887	157.87	5.25E + 11
0.60	y = −14395x + 21.333	0.9813	119.68	2.03E + 08
0.65	y = −10742x + 15.022	0.9657	89.31	3.65E + 05
0.70	y = −15536x + 21.579	0.9878	129.17	1.44E + 09
**Average**		**0.9854**	**132.03**	**8.11E + 10**
**Flynn–Wall–Ozawa method**				
0.10	y = −9463.7x + 20.702	0.9869	74.79	1.71E + 04
0.15	y = −13993x + 28.077	0.9926	110.59	3.08E + 07
0.20	y = −15222x + 29.708	0.9979	120.30	2.31E + 08
0.25	y = −16296x + 31.168	0.9910	128.79	1.33E + 09
0.30	y = −16226x + 30.666	0.9981	128.23	1.19E + 09
0.35	y = −16540x + 30.871	0.9985	130.72	1.98E + 09
0.40	y = −17355x + 31.951	0.9941	137.16	7.46E + 09
0.45	y = −17915x + 32.609	0.9897	141.58	1.85E + 10
0.50	y = −17793x + 32.141	0.9942	140.62	1.52E + 10
0.55	y = −17108x + 30.759	0.9975	135.21	4.99E + 09
0.60	y = −17094x + 30.485	0.9915	135.09	4.88E + 09
0.65	y = −14206x + 25.514	0.9944	112.27	4.37E + 07
0.70	y = −10898x + 19.913	0.9929	86.13	1.87E + 05
**Average**		**0.9938**	**121.65**	**4.30E + 09**
**Kissinger–Akahira–Sunose method**				
0.10	y = −8406.1x + 6.1598	0.9836	69.89	6.05E + 03
0.15	y = −12885x + 13.443	0.9914	107.13	1.50E + 07
0.20	y = −14089x + 15.029	0.9975	117.14	1.20E + 08
0.25	y = −15146x + 16.459	0.9897	125.92	7.37E + 08
0.30	y = −15060x + 15.929	0.9978	125.21	6.36E + 08
0.35	y = −15360x + 16.110	0.9983	127.70	1.06E + 09
0.40	y = −16163x + 17.171	0.9933	134.38	4.21E + 09
0.45	y = −16713x + 17.811	0.9883	138.95	1.08E + 10
0.50	y = −16579x + 17.324	0.9934	137.84	8.60E + 09
0.55	y = −15883x + 15.923	0.9972	132.05	2.61E + 09
0.60	y = −15859x + 15.633	0.9902	131.85	2.50E + 09
0.65	y = −12954x + 10.635	0.9934	107.70	1.69E + 07
0.70	y = −9622.7x + 4.996	0.9911	80.00	5.16E + 04
**Average**		**0.9865**	**118.14**	**2.41E + 09**
**Kissinger method**				
**α not involved**	y = −18746x + 20.857	**0.9506**	**155.85**	**3.47E + 11**

**Table 5:** Reaction order (*n*) deduced from Avrami’s theory for *C. aesculifolia*.

T (K)	Slope (*m*) of the Equation	Reaction Order (*n* = −*m*)	Correlation Coefficient (*R*^2^)
500	−0.4774	0.4774	0.9531
525	−0.3937	0.3937	0.9986
550	−0.5114	0.5114	0.9976
575	−0.6141	0.6141	0.9992
600	−0.5890	0.5890	0.9986
625	−0.4456	0.4456	0.9889
650	−0.3895	0.3895	0.9963
**Average**		**0.4887**	**0.9903**

**Table 6:** Thermodynamic properties of *C. aesculifolia*.

	Friedman			FWO			KAS		
α	ΔH	ΔG	ΔS	ΔH	ΔG	ΔS	ΔH	ΔG	ΔS
0.10	77.51	177.21	−164.51	70.41	177.67	−176.98	65.51	178.01	−185.63
0.15	145.43	174.16	−47.41	106.00	175.69	−115.01	102.53	175.85	−120.99
0.20	126.56	174.83	−79.66	115.61	175.27	−98.45	112.45	175.40	−103.89
0.25	117.96	175.17	−94.40	124.03	174.93	−84.00	121.16	175.04	−88.91
0.30	131.97	174.62	−70.38	123.41	174.95	−85.05	120.38	175.07	−90.24
0.35	143.84	174.20	−50.11	125.83	174.85	−80.89	122.82	174.97	−86.06
0.40	148.16	174.06	−42.73	132.22	174.61	−69.95	129.44	174.71	−74.71
0.45	142.57	174.24	−52.26	136.60	174.45	−62.46	133.96	174.54	−66.96
0.50	143.23	174.22	−51.13	135.59	174.48	−64.19	132.81	174.58	−68.93
0.55	152.80	173.90	−34.82	130.13	174.68	−73.52	126.97	174.80	−78.92
0.60	114.55	175.30	−100.23	129.97	174.69	−73.79	126.73	174.81	−79.34
0.65	84.12	176.77	−152.89	107.08	175.62	−113.09	102.51	175.83	−120.98
0.70	123.89	174.91	−84.20	80.85	176.95	−158.59	74.72	177.33	−169.31
**Average**	**127.12**	**174.89**	**−78.83**	**116.75**	**175.30**	**−96.61**	**113.23**	**175.46**	**−102.68**
**Kissinger**	ΔH			ΔG			ΔS		
	**150.95**			**173.97**			**−37.98**		

ΔH and ΔG are given in kJ/mol, and ΔS in J/mol. K.

**Table 7:** Characteristics of *C. aesculifolia* trees sampled.

Trees	Geographic Location	DBH (cm) ^1^	Tree Height (m)	SLHA (m) ^2^
1	19°58′37″N, 101°5′42″O	16	5.30	1850
2	19°58′13″N, 101°5′38″O	23	8.50	1940
3	19°58′18″N, 101°6′3″O	17	5.40	1920

^1^ Diameter at breast height. ^2^ Height above sea level.

## Data Availability

All data supporting the reported results are available upon request from the corresponding author.
